# Identification and Categorization of the Top 100 Articles and the Future of Large Language Models: Thematic Analysis Using Bibliometric Analysis

**DOI:** 10.2196/68603

**Published:** 2025-08-27

**Authors:** Ethan Bernstein, Anya Ramsamooj, Kelsey L Millar, Zachary C Lum

**Affiliations:** 1 College of Medicine, California Northstate University, Elk Grove, CA, United States; 2 Department of Orthopaedic Surgery, University of California Davis Medical Center, 4860 Y Street, Suite 1700, Sacramento, CA, 95817, United States, 1 (916) 7342700, 1 (916) 7347137

**Keywords:** large language models, ChatGPT, Web of Science, medicine, education, technology, research categorization, artificial intelligence

## Abstract

**Background:**

Since the release of ChatGPT and other large language models (LLMs), there has been a significant increase in academic publications exploring their capabilities and implications across various fields, such as medicine, education, and technology.

**Objective:**

This study aims to identify the most influential academic works on LLMs published in the past year, categorize their research types and thematic focuses, within different professional fields. The study also evaluates the ability of artificial intelligence (AI) tools, such as ChatGPT, to accurately classify academic research.

**Methods:**

We conducted a bibliometric analysis using Clarivate’s Web of Science (WOS) to extract the top 100 most cited papers on LLMs. Papers were manually categorized by field, journal, author, and research type. ChatGPT-4 was used to generate categorizations for the same papers, and its performance was compared to human classifications. We summarized the distribution of research fields and assessed the concordance between AI-generated and manual classifications.

**Results:**

Medicine emerged as the predominant field among the top 100 most cited papers, accounting for 43 (43%), followed by education 26 (26%) and technology 15 (15%). Medical literature primarily focused on clinical applications of LLMs, limitations of AI in health care, and the role of AI in medical education. In education, research was centered around ethical concerns and potential applications of AI for teaching and learning. ChatGPT demonstrated variable concordance with human reviewers, achieving an agreement rating of 47% for research types and 92% for fields of study.

**Conclusions:**

While LLMs such as ChatGPT exhibit considerable potential in aiding research categorization, human oversight remains essential to address issues such as hallucinations, outdated information, and biases in AI-generated outputs. This study highlights the transformative potential of LLMs across multiple sectors and emphasizes the importance of continuous ethical evaluation and iterative improvement of AI systems to maximize their benefits while minimizing risks.

## Introduction

Within academic literature, artificial intelligence (AI) is broadly defined as a mechanical emulation of the human thinking processes to facilitate the analysis, simulation, exploitation, and exploration of human thinking processes [1]. ChatGPT has been trained on a massive amount of internet data from 2021 and is being updated from here on to include current data and information retrieval from the internet, which should reflect current, up-to-date information. It uses deep neural networks, machine learning, and a training dataset to interact with prompts and generate relevant human-like text responses [2], qualifying it as a large language model (LLM) [3]. ChatGPT may have significant potential to improve the efficiency of human innovation in a multitude of fields that require quick access to information, such as medicine and education, by providing instant feedback and helping to expedite clerical work, such as writing notes or research processes.

LLMs have been a topic of interest for researchers in many fields, with prior bibliometric analyses finding there to be an increase from 19 papers in 2017 to 2486 papers on LLMs as of 2023. The primary topics of studies published at that time were the utility of LLMs and the fields of interest where the use of LLMs could be implemented [4]. Studies focused on just ChatGPT rather than LLMs as a whole and identified the most influential authors and countries for research on ChatGPT and tracing the rapid evolution of ChatGPT scholarship [5]. More recently, a bibliometric analysis in 2025 similarly identified the most productive institutions, in addition to countries and authors [6]. Identifying the most productive institutions, geographical regions, and authors in LLM research has been one of the major topics of interest with the overarching goal of pinpointing the best potential uses for LLMs in their respective fields of study. Another bibliometric analysis published in 2024 used Scopus to identify 82 publications on ChatGPT in educational research. They found that the *Journal of University Teaching and Learning Practice* had published the most papers on this topic and identified the most cited publications in the field of education. Common areas of study included benefits and uses, academic writing, and best practices. They cited the timing of their research and their search parameters only including English articles in their screening as limitations. These bibliometric trends highlight the exponential growth and global interest in LLM-related research, particularly around ChatGPT, and highlight a shift from broad explorations of utility toward more targeted analyses of influence, productivity, and practical applications.

It has been over a year since the public release of ChatGPT by the company OpenAI, followed by Gemini (formerly BARD) by Google and subsequent widespread use of AI, from usage by students for writing support to researchers for literature review and manuscript preparation assistance. From its release through August 2023, there have been over 1000 PubMed citations [7], indicating its rapid rise in use and interest in its capabilities. With any new technology, many use cases are developed and tested until a narrowing of the field emerges. This determines which aspects of technology resonate within the community and which aspects need further refinement. We sought to determine which types of studies were performed, which fields of research have the most studies, and which journals are the most highly cited, as this may highlight fields that are the most intense focus of researchers regarding the use of AI. We also sought to determine how well ChatGPT performs thematic categorization of research type and field of research when analyzing academic publications.

## Methods

### Overview

We used Clarivate Web of Science (WOS) and searched for all research articles with the terms “chatgpt,” “bard,” and “large language model” independently in March 2024. Papers with the 100 highest citation counts were exported from WOS into a spreadsheet and categorized by journal, author, research type, and field of study by 2 authors (EB and AR). Any discrepancies were resolved by a third reviewer. We also used ChatGPT-4 (GPT-4 model) to thematically categorize the papers for comparison with the manual grouping related to AI. ChatGPT was chosen for thematic categorization due to its popularity, with 89/100 papers explicitly mentioning ChatGPT in the title and 98/100 discussing or using ChatGPT in their studies, and its superior performance in Answer-Only settings and static data compared to other LLMs [8]. The literature review was conducted from June to July 2024.

### Author’s Categorization

Python (version 3.11.7; Python Software Foundation) was used to count the number of times an author was listed in the top 100 cited publications in the Excel spreadsheet generated by WOS and rank the authors according to frequency. Python was also used to count the number of times a journal was listed in the top 100 cited publications and rank in order of frequency. This code is depicted in section 2a-b in Multimedia Appendix 1. Section 4 in Multimedia Appendix 1 was generated via Python.

Based on a review of each study’s abstract, the papers were categorized by the general field of research in which the study was conducted and the type of research conducted, which was counted manually in an Excel (Microsoft Corp) spreadsheet. Some papers could be categorized under the purview of multiple fields; for example, papers discussing medical education could have been categorized as either medicine or education. In such cases, the primary field of focus of the publishing journal was referred to for determination. These tasks were carried out by 2 authors (EMB and AR), with any discrepancies reconciled by a third party. A 2+1 independent observer model was implemented to reduce potential misclassification bias. The type of research was determined and recorded for each study, and Python was used to count and sort by frequency (section 2c in Multimedia Appendix 1). Ranganathan and Aggarwal [9] were referred to for the research types, which were expanded by the authors to include a more comprehensive list of research types. These categories are listed in the “Results” section.

### Literature Review

Following the categorization stage, the most cited areas of research were determined, and a full literature review was performed of the included papers. In total, 84 papers were included in the literature review; 16 papers were excluded due to the lack of similar content that made it difficult to draw meaningful and cohesive conclusions. Each paper was analyzed to determine the primary topics of interest regarding AI and LLMs. A list of topics was generated as each paper was read, and the number of papers that covered each topic was recorded in a spreadsheet. For example, if a paper in the medicine category covered the clinical uses of ChatGPT, it was marked and counted toward the total number of papers that covered that topic. This was done by 2 authors (EMB and AR) performing the literature review, and any discrepancies were reconciled by discussion between the 2 reviewers. These areas are further discussed in the “Results” section of this paper. The goal of this study is to explore the fields that are most interested in AI LLMs and assess current and future implications of AI LLMs in each respective field. In addition, we aimed to evaluate ChatGPT’s ability to analyze and categorize scientific literature.

### Thematic Categorization Performed by ChatGPT (GPT-4)

ChatGPT (GPT-4 model) was asked to count and report authors and journals in order of frequency. The full list of authors and journals was pasted into ChatGPT and ChatGPT was prompted to sort by frequency. These generated lists were then compared to the corresponding lists generated by the authors.

ChatGPT was also asked to determine author, journal, research type, and field of research. A PDF copy of each paper was uploaded to ChatGPT, which was asked to extract the author and journal and to determine the study type and field of research. A set list of study types, which was used by the authors for research type categorization, was added to the prompts as there are many possibilities for this output. The prompts in ChatGPT were generated by one author (EMB). ChatGPT results were then directly compared to the results generated by the corresponding author to assess accuracy. To minimize potential biases from prompts provided by the author and to record ChatGPT’s responses for research classification most authentically, it was not pre-trained, as daily users may not use a pretraining process, or require an extensively trained version of ChatGPT. ChatGPT’s categorization for research type was compared to the author’s categorization. All extractions were performed in a new thread to reduce hallucinations. Examples of these prompts are listed in section 3 in Multimedia Appendix 1.

### Ethical Considerations

This study did not involve human or animal participants. Institutional review board approval, informed consent, data confidentiality, and participant compensation were not applicable.

## Results

### Publication Sources

Overall, there were 12 authors whose names appeared twice in the top 100 citations, but none appeared more than twice. Across the top 100 cited papers, the *Cureus Journal of Medical Science* had the most, with 7 papers (5-year Impact Factor of 1.1, with a multispecialty subject area). *Educational Sciences* and *Journal of Medical Internet Research* each had 3 papers (5-year Impact Factors of 2.6 and 6.7, and educational and health informatics subject areas, respectively). Every other journal had either 1 or 2 publications on the top 100 cited papers list (section 4 in Multimedia Appendix 1).

### Field of Research

Medicine was the most frequently cited field with 43 papers, followed by education and educational research with 26 papers and technology and information sciences with 15 papers. Less frequently mentioned categories included business and economics, and tourism, both of which had 4 papers. All other areas had either 1 or 2 papers in the top 100. These findings are summarized in Table 1 with further breakdown of individual categorizations in sections 5-7 in Multimedia Appendix 1.

Multiple medical subfields have published papers regarding ChatGPT. General and internal medicine was the most mentioned subcategory with 11 publications, followed by health care sciences and services with 7, surgery with 5, oncology with 4, radiology with 3, and ophthalmology with 3. All other areas of medicine had only 1 publication mentioned. These findings are summarized in Table 2.

**Table 1.** Areas of research as generated by the author (EMB).

Area of study	Publications (N=100)
Medicine, n (%)	43^a^ (43)
Education, n (%)	26^a^ (26)
Technology and information sciences, n (%)	15^a^ (15)
Business and economics, n (%)	4 (4)
Tourism, n (%)	4 (4)
Government and law, n (%)	2 (2)
Public health, n (%)	2 (2)
Basic sciences, n (%)	1 (1)
Ethics, n (%)	1 (1)
Geography, n (%)	1 (1)
Pharmacology, n (%)	1 (1)

a Top 3 areas of research included in literature review.

**Table 2.** Breakdown of specific fields of medicine in which papers were published.

Field of Medicine	Papers
General and internal medicine, n (%)	11 (26)
Health care sciences and services, n (%)	8 (19)
Surgery, n (%)	5 (12)
Oncology, n (%)	4 (9)
Radiology, nuclear medicine, and medical imaging, n (%)	3 (7)
Ophthalmology, n (%)	3 (7)
Nursing, n (%)	1 (2)
Gastroenterology and hepatology, n (%)	1 (2)
Endocrinology and metabolism, n (%)	1 (2)
Otorhinolaryngology, n (%)	1 (2)
Medical ethics, n (%)	1 (2)
Sport sciences, n (%)	1 (2)
Dentistry, oral surgery, and medicine, n (%)	1 (2)
Biomedical social sciences, n (%)	1 (2)
Nursing, n (%)	1 (2)
Total number of papers in medicine, n	43

### Research Type

A total of 25 descriptive analyses were included, followed by 23 narrative reviews, 17 analytical observational studies, 16 opinion or editorial papers, 11 theoretical or conceptual papers, 4 systematic reviews, 3 mixed methods studies, and 1 analytical interventional study.

Descriptive studies, analytical observational, analytical interventional, systematic review, and meta-analysis were defined in Ranganathan and Aggarwal [9]. This list was expanded to include narrative reviews, which were defined as an overview of a current topic without the use of inclusion and exclusion criteria for article identification. Case reports and case series were defined as real-life use cases in a practical or clinical setting. Opinion, Editorial, or Perspective papers included those that discussed a topic and the author’s personal view or opinion on a topic but did not perform any study or statistical analysis. Theoretical or conceptual papers discussed potential uses of LLMs but did not provide real-life examples or experimentation with LLMs. Mixed methods papers used qualitative and quantitative evidence.

### Medicine

Of the 43 papers [7,10-46] reviewed from the field of medicine, 38 (88%) papers discussed the limitations of ChatGPT, 30 (70%) discussed the uses of ChatGPT in clinical medicine, 21 (49%) numerically evaluated the quality and capability of ChatGPT in regards to medical reasoning, and 9 (21%) evaluated the uses of ChatGPT in medical research. Medical education was an additional area of focus with 14 (33%) papers discussing ChatGPT’s ability to answer board certification questions or help with studying from both the student and educator perspective. A total of 19 (44%) papers discussed the uses in research and 18 (42%) discussed ethical considerations.

Studies that covered quality assessment in medical knowledge evaluated the accuracy of ChatGPT’s answer to specific medical questions from patients and board examination questions and the capability to use higher-order medical reasoning. Many of these papers cited promising results and indicated that further research and technology improvements are necessary prior to full implementation into the medical field. Many papers discussed the already proven or hypothetical uses of ChatGPT in medicine, such as the enhancement of telemedicine, answering patient questions, and administrative tasks such as charting and other paperwork [10,11]. Potential weaknesses preventing current widespread adoption included lack of nuanced information that an experienced physician would understand and potential inaccuracies due to biased or outdated training data [12,13]. Authors cited the high level of confidence that ChatGPT appears to answer with, which may result in the dissemination of misinformation [14]. Further improvement of the technology would be necessary due to lack of deep understanding and inability to interpret complex medical imaging. Some studies compared physician responses to AI responses and found that evaluators may prefer the AI-generated response.

Other uses in medicine included assistance for nonnative English speakers with translation to their native language [15-17]. AI is able to quickly comprehend information, which indicates a potential use for providing information about medical guidelines in acute situations, such as in the intensive care unit [10]. AI may also be useful in assisting with documentation, decision support, and patient communication.

Quality assessment in research discussed the automatic generation of citations, manuscripts, ideas, and hypotheses. The ability of ChatGPT and other LLMs to conduct literature searches was also evaluated [37]. Some studies synthesized the already proven uses in research, the most prominent of which included literature review, data synthesis, and assistance with data analysis [10,47]. Review papers covering research utility discussed the ability to generate large amounts of text and can help authors organize their thoughts [21]. ChatGPT has been shown to quickly summarize whole papers well and can save researchers significant amounts of time [48].

Limitations of AI models were the most commonly discussed topic, with many papers referencing lack of updated information because ChatGPT was trained on data from 2021, which may lead to outdated or incorrect information. The possibility of ChatGPT hallucination, generation of fake citations, and perpetuation of biases contained in the information that it was trained on was also discussed [15,17]. Papers that discussed ethics included privacy concerns with cybersecurity risks with patient confidentiality as ChatGPT stores its conversations in its memory bank with minimal evidence that it can comply with Health Insurance Portability and Accountability Act (HIPAA) regulations [24]. One paper reported concerns with shortcuts in the research or learning process, which could lead to inflated numbers of publications without the same level of expertise [21].

Papers regarding medical education discussed potential use via interactive simulation, immediate feedback and information, and creation of educational materials for instructors. Students can generate quiz questions, which can alleviate the work burden for educators and students alike. Studies such as Huh [49] showed that ChatGPT performed comparably to medical students in certain assessments indicating the potential for integration and use in medical education. Multiple studies cited promising results of ChatGPT answering board questions.

### Education

A total of 26 papers [49-74] were categorized as education as their field of study. Of these, 21 (81%) education papers discussed the ethical concerns associated with AI implementation in schools and 3 (12%) discussed privacy considerations; 7 (27%) discussed the potential negative implications on students’ learning and performance capabilities, and 17 (65%) discussed solutions for these concerns. In total, 18 (69%) papers discussed the potential implementations for students and 17 (65%) discussed potential implementations for educators. In addition, 9 (35%) performed a quality assessment, 18 (69%) discussed the capabilities of AI, and 18 (69%) discussed limitations.

In the field of education, the main privacy concerns were related to the storage of student data and personal information that may be stored by ChatGPT through regular usage. Papers discussed the importance of data security and compliance with privacy regulations to prevent personal information from being disseminated.

Education ethics primarily discussed academic dishonesty including plagiarism and incorrect citations. Evidence advocating for ChatGPT as a reliable author or primary source is minimal, and the academic ethical implications of copying and pasting the ChatGPT response were unclear; however, the consensus questioned the ethics of directly quoting ChatGPT without attributing it as the source [50,75]. Additional sources of ethical concerns were regarding the discordant access to ChatGPT, as the optimized version is currently a paid service [51,52]. The perpetuation of discriminatory and biased ideas in the information that ChatGPT was trained on was also a common concern, which led papers to recommend cautious use of AI and special care to identify and mitigate such biases [22,53]. Educators have expressed concerns over students' overreliance leading to loss of writing skills and hindrance of creativity in addition to academic dishonesty.

Solutions to AI overreliance included the implementation of AI literacy early in the education system, similar to how typing and technology training became ubiquitous with the rise of computers [54]. Other workarounds to overreliance on ChatGPT include assignment design that is incompatible with ChatGPT [55,56]. Educators are being urged to specify the permitted usage of ChatGPT in their course syllabi for transparency [57]. On the side of OpenAI, regular updates to ensure the accuracy of information available to ChatGPT are also crucial. Perkins [57] discussed the need for transparency and guidelines for AI use in academic settings.

Quality assessments evaluated accuracy, relevance, and potential biases of text generated by ChatGPT in multiple educational fields generally, or within specific areas such as chemistry, language, administration, and academic research. Researchers investigated the capability to enhance learning, teaching, and grading. Fergus et al [58] evaluated ChatGPT’s ability to generate assessment questions in chemistry and the quality of the answers generated by ChatGPT. Farrokhnia et al [59] performed a SWOT (Strengths, Weaknesses, Opportunities, and Threats) analysis of ChatGPT in education and research, reporting the ability to provide real-time feedback and grading with specific responses, which can decrease workload for both students and educators. Other papers explored academic integrity and third-party programs’ ability to detect work written by ChatGPT, the ability of ChatGPT to generate educational materials, such as textbooks, study guides, and practice questions, and the ability for AI to assist students with learning experience and motivation. Papers marked under uses for students and uses for educators included relevant quality assessments and review papers covering proven uses.

ChatGPT’s limitations in education include its potential for generating inaccurate or biased information, its dependency on up-to-date training data, and its inability to fully replace human educators [62,64]. For example, while ChatGPT can provide factual information, it lacks the ability to engage in nuanced discussions or provide emotional support [52].

### Technology and Information Sciences

A total of 15 papers [3,47,76-87] were categorized as reviewed technology and information sciences. Subtopics included computer science, engineering and electric vehicles, and data science. Of these, 12 (80%) papers discussed the technical uses of ChatGPT, with 2 (13%) of those being quality assessment papers. In addition, 6 (40%) discussed ethics and privacy concerns, and 9 (60%) discussed limitations. Only 1 (7%) discussed the public perception of AI and 9 (60%) discussed the future applications and implementations of AI in the workplace and daily life.

Ethics in the field of technology and information sciences included privacy concerns with the handling and storage of user data. Like other sections, authors emphasized the importance of strong cybersecurity measures including encryption and secure storage to minimize the risk with security breaches. Uniquely, some papers discussed the potential of AI to create harmful “deep fake” content, which has the potential to be weaponized [76,88]. Deep fake is defined as high-quality fabricated image and video content that can be misinterpreted by viewers as being real [89].

Technical uses of ChatGPT in this field included code generation, debugging, and automated routine IT processes. The ability to assist with data science through data cleaning, preprocessing, and preliminary result interpretation was explored [77,78]. Du et al [79] discussed the potential of AI implementation in electric vehicles. Holzinger et al [90] covered AI from the biotechnology perspective, which provided an overview of the uses of AI to agricultural engineering, medical biotechnology, and bioinformatics. AI is already being used by plant tissue scientists to simulate complex interactions and treatment options for their agricultural experimentation. AI can benefit medicine from a microperspective, including genomic analysis, biomedical image analysis, data analytics, and drug discovery and development [90]. The advantage comes from the ability to rapidly analyze large quantities of data through automation and the implementation of predictive models that can analyze image data and recommend the best management and planning course for any given task. Quality assessment papers that analyzed potential uses of ChatGPT looked at AI’s writing abilities in order to contribute to the conversation surrounding job security [91].

Limitations of AI in the field of technology and information sciences include difficulty with performance in complex problem-solving scenarios, outdated training data, and potential inaccuracies [77]. Authors emphasized that AI requires human oversight to ensure reliable outputs [80]. For example, while ChatGPT can assist with coding, it might not always understand the context of complex software projects, leading to errors [85]. Future directions that the academic technology community is hoping ChatGPT and other AI models take include improvement of privacy concerns and continued efforts to address ethical concerns [76]. In addition, improving reliability to decrease the likelihood of hallucinations and increasing complex understanding to improve usability and reliability [79].

Finally, one paper examined public perception of ChatGPT by evaluating responses on X (previously Twitter; X Corp) to determine generally how internet users felt about the dawn of AI [84].

### ChatGPT’s Thematic Categorization

When asked to report the frequency of authors mentioned out of the top 100, ChatGPT generated a different list than the manually generated list of author frequency, which was confirmed to be incorrect upon verification. It was also unable to correctly count the frequency of each journal. For example, it counted 11 occurrences in the *Cureus Journal of Medical Science* and 4 occurrences in the *Journal of Medical Internet Research*. The manual extraction yielded 7 occurrences in the *Cureus Journal of Medical Science* and 3 occurrences in the *Journal of Medical Internet Research*. ChatGPT’s outputs for these categories are included in Section 8 in Multimedia Appendix 1. ChatGPT identified the research type correctly in 86% of cases. The field of research was correctly identified by ChatGPT only 47% of the time. In some cases, ChatGPT was asked to reconsider its categorization and often changed its determination when prompted by the user. It was highly susceptible to hallucinating information for the wrong paper when used in a single thread. To minimize these hallucinations, a new thread had to be made for each paper’s analysis. Percentages reflect a simple ratio of matching results to total results.

## Discussion

### Principal Findings

This discussion explores the current trajectory of AI integration, potential breakthroughs, and the implications for the following fields.

### Medicine

In medicine, AI is primarily being tested to assist in areas from administrative support to clinical applications. The potential of AI to enhance telemedicine, streamline administrative tasks, and assist in diagnostic processes is well documented [11,22,53]. However, the accuracy and reliability of AI in medical decision-making are areas that require further research. Future pathways include the integration of AI into clinical workflows to assist with patient triage, diagnostic support, and personalized treatment plans [11]. The field appears to be moving toward leveraging AI to augment, rather than replace, human expertise, with potential breakthroughs anticipated in predictive analytics and personalized medicine [16,37,46,92]. The ongoing challenge will be ensuring AI systems are trained on up-to-date and diverse datasets to minimize bias and inaccuracies. Before widespread integration of AI in medicine occurs, further research is necessary to prove reliability and improvement from early models [22].

In addition, prior research suggests that programs such as ChatGPT can assist with medical education. For instance, Huh [49] demonstrated that ChatGPT, while not outperforming medical students, provided reasonable answers on parasitology examinations, indicating its potential for educational integration. The literature also indicates that research processes are being disrupted, with AI showing promise in tasks such as automatic generation of citations, manuscripts, and hypotheses, which can streamline the research process [50]. However, reducing the hallucination frequency and improving accuracy will need to be made before any significant disruption in clinical practice can occur.

### Education

Education has the potential to enter a transformative phase with the incorporation of AI tools such as ChatGPT into current curricula. These tools can offer substantial benefits, including personalized learning experiences, automated grading, and enhanced teaching aids. However, the potential for academic dishonesty and the ethical implications of AI use in education remain significant concerns [48,53,54,59,67,71]. As AI technology improves, it is expected to offer even more sophisticated support for both students and educators, such as adaptive learning platforms that cater to individual student needs and real-time feedback systems. The field is also exploring AI’s role in reducing teacher workload and providing continuous professional development opportunities [48,51,59,67]. Ensuring equitable access to AI tools and addressing ethical dilemmas will be crucial as AI becomes more integrated into educational systems.

### Technology and Information Sciences

AI’s impact on technology and information sciences, particularly in software development, data analysis, and cybersecurity, has positive indications for progress. AI tools are increasingly used for code generation, something that was successfully implemented in this study to help with counting author, journal, study type frequencies, and generating figures. Debugging, automating routine IT tasks, and enhancing productivity and efficiency are other capabilities [3,82,85]. Future breakthroughs are expected in the development of more reliable and context-aware AI systems capable of handling complex problem-solving scenarios. Ethical concerns, such as protecting privacy and security, will continue to be pivotal, with ongoing efforts to develop robust cybersecurity measures and ethical AI guidelines.

### Thematic Categorization

The results of ChatGPT’s thematic categorization suggest that further improvements must be made before ChatGPT can reliably sort and organize data. At the time the study was performed, it appeared as though ChatGPT was overwhelmed by large amounts of text, which raises questions about its capability to sort through information such as lists of names and journals; when asked to sort through the list of authors, which contained over 190 names, the sorted list was incorrect. It was unreliable in determining the study type. However, it did perform well in determining the field of research, suggesting that ChatGPT can be trusted with simpler and more straightforward tasks.

### Limitations

Limitations of this study include the large breadth of research study types and similarities between different study types that each paper could have been classified as, leading to potential difficulties reproducing similar results in future similar studies. ChatGPT and research on AI models are always rapidly developing, making the likelihood that some conclusions drawn may have newer, updated information. There are also legal and ethical factors to consider when uploading copies of research papers to ChatGPT. At the time of writing, there are no explicit laws or journal terms of service that prohibit uploading PDF copies to ChatGPT, making this an area of legal ambiguity. Each paper was legally downloaded and used solely for private purposes. No research was redistributed, and there was no commercial benefit from using these papers. Notably, 75% of the papers were open access. Under fair use, the content was used privately and was not used for commercial research, redistribution, reproduction, or sale. However, as ChatGPT continues to grow in popularity and utility, academic institutions, publishers, and developers will need to reassess the ethical and legal boundaries of uploading copyrighted material to these tools. In addition, there are long-term memory storage issues, as it can only store a limited set of facts or preferences. This makes using singular threads extracting information more likely to hallucinate incorrect information, as ChatGPT may provide incorrect information rather than admitting it does not know the answer. It may require frequent prompting for optimal performance. Methods such as frequently generating new threads to prevent overloading information stored in long-term memory can be effective for avoiding this problem, as used in this study.

### Future Directions

In the context of this study, ChatGPT should be improved to better aid in thematic categorization. Thematic categorization of the field of research sometimes required information that was not directly stated in the paper, which would make it very difficult for ChatGPT to correctly determine the field of research. For example, some papers covered technology topics but were published in a medical journal, resulting in incorrect categorization as technology, rather than medicine [45]. This discrepancy may be attributed to author criteria rather than technology failure; however, it indicates the importance of providing detailed, specific instructions to LLMs in order to receive the desired output. It is important to acknowledge that ChatGPT continues to have difficulties with hallucination and persistent long-term memory and limited context retention, which can impair usability and reliability for users. Due to such limitations, users should monitor the outputs and verify accuracy. Broadly, future research should focus on refining ChatGPT to alleviate any privacy concerns, hallucinations, and bias.

### Conclusions

Medicine, education, and technology are preparing for a future with potential LLM integration, as demonstrated by the high citation counts in these fields. While LLMs such as ChatGPT offer promise in streamlining workflows and categorizing research, this study underscores the importance of human oversight to address risks such as hallucination, outdated information, and bias. Realizing AI’s full potential will require responsible implementation that supports human expertise, ensures equitable access, and maintains up-to-date information. In medicine, AI is expected to integrate further into clinical workflows, assisting with diagnostics, patient communication, and administrative tasks like charting, although concerns remain about accuracy and ethical implications. In education, AI tools may be able to revolutionize personalized learning, automate grading, and support educators, but addressing academic dishonesty and ensuring ethical AI use in learning environments is crucial. In technology, LLMs are advancing software development, data science, and cybersecurity, but future work needs to enhance AI’s ability to handle complex problem-solving while ensuring privacy and security.

These fields are tightly linked, with educational pedagogy aiding the development of physicians, and the field of technology developing the software and apps that physicians will use. If AI positively affects education, it may result in a network of physicians who can use advanced technology to increase efficiency in practice and improve patient care. Furthermore, AI is being developed to enhance research processes, providing resources for researchers to improve productivity, output, and in effect, impact. LLMs are moving toward transforming the efficiency and quality of these fields through continued improvement and integration. Ultimately, the true potential of AI can be realized through a collaborative approach, where human expertise works in tandem with AI, ensuring that both ethics and efficiency are upheld.

## Supplementary material

10.2196/68603 Multimedia Appendix 1 Contains data collected and links to specific ChatGPT threads used to perform the study. Includes items from 8 sections.

## Abbreviations

AI: artificial intelligence
HIPAA: Health Insurance Portability and Accountability Act
LLM: large language model
SWOT: Strengths, Weaknesses, Opportunities, and Threats
WOS: Web of Science
