# Supplementary material for: Identification and Categorization of the Top 100 Articles and the Future of Large Language Models: Thematic Analysis Using Bibliometric Analysis
Source: JMIR AI. 2025 Aug 27;4:e68603. doi: 10.2196/68603 (PMC12384689; doi:10.2196/68603)
Supplement: Multimedia Appendix 1 [file ai-v4-e68603-s001.docx]

**Section 1. Search Strategy**

Clarivate Web of Science (WOS) was searched for all research articles with the terms “chatgpt” and “bard” and “large language model” independently. The 100 articles with the highest citation numbers were chosen for this analysis.

**Section 2a-b. Python code**

a) Python code to count the number of times a journal name is mentioned in an Excel spreadsheet and then output the results from most to least mentioned.
b) Python code to count the number of authors and organize from most mentioned to least mentioned.

**
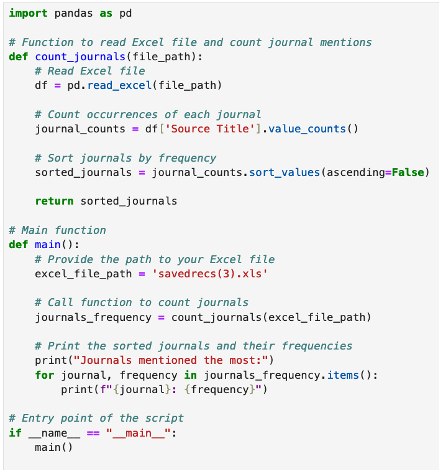

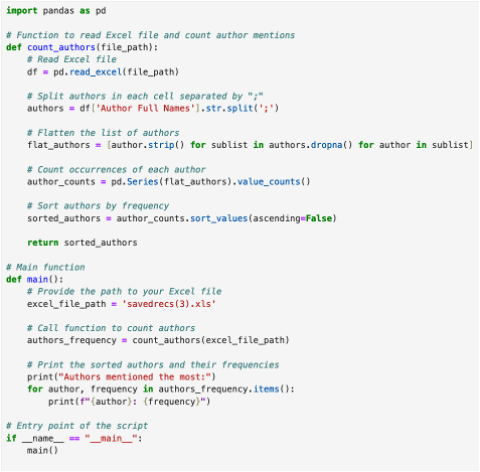
**

**Section 2c. Python code**

c) Python code to count the number of study types mentioned and generate a bar graph.

**
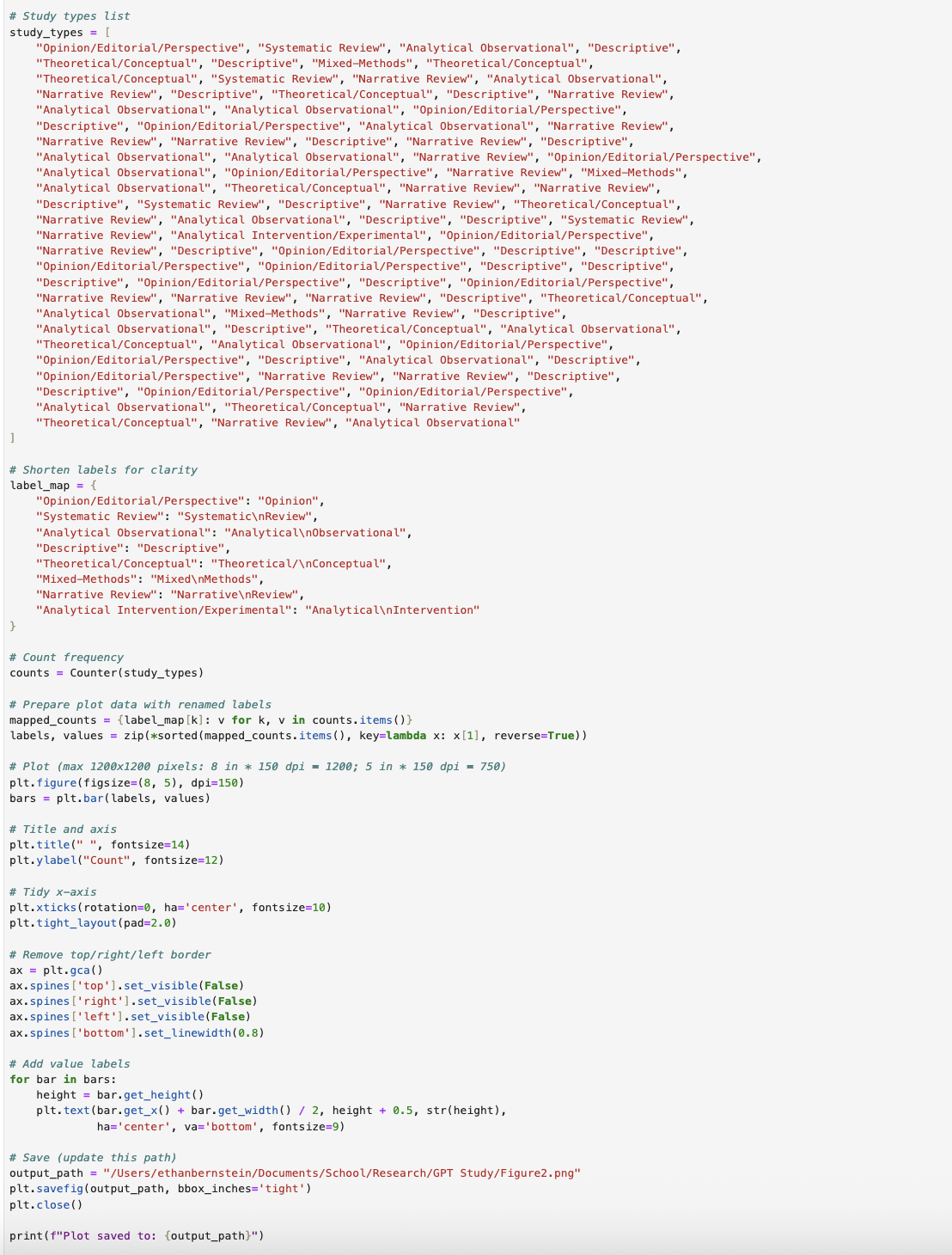
**

**Section 3. Thematic categorization text thread prompts.**

[**https://chatgpt.com/share/fc4dcb45-9285-413b-98a0-6108d6208844/continue**](https://chatgpt.com/share/fc4dcb45-9285-413b-98a0-6108d6208844/continue)**
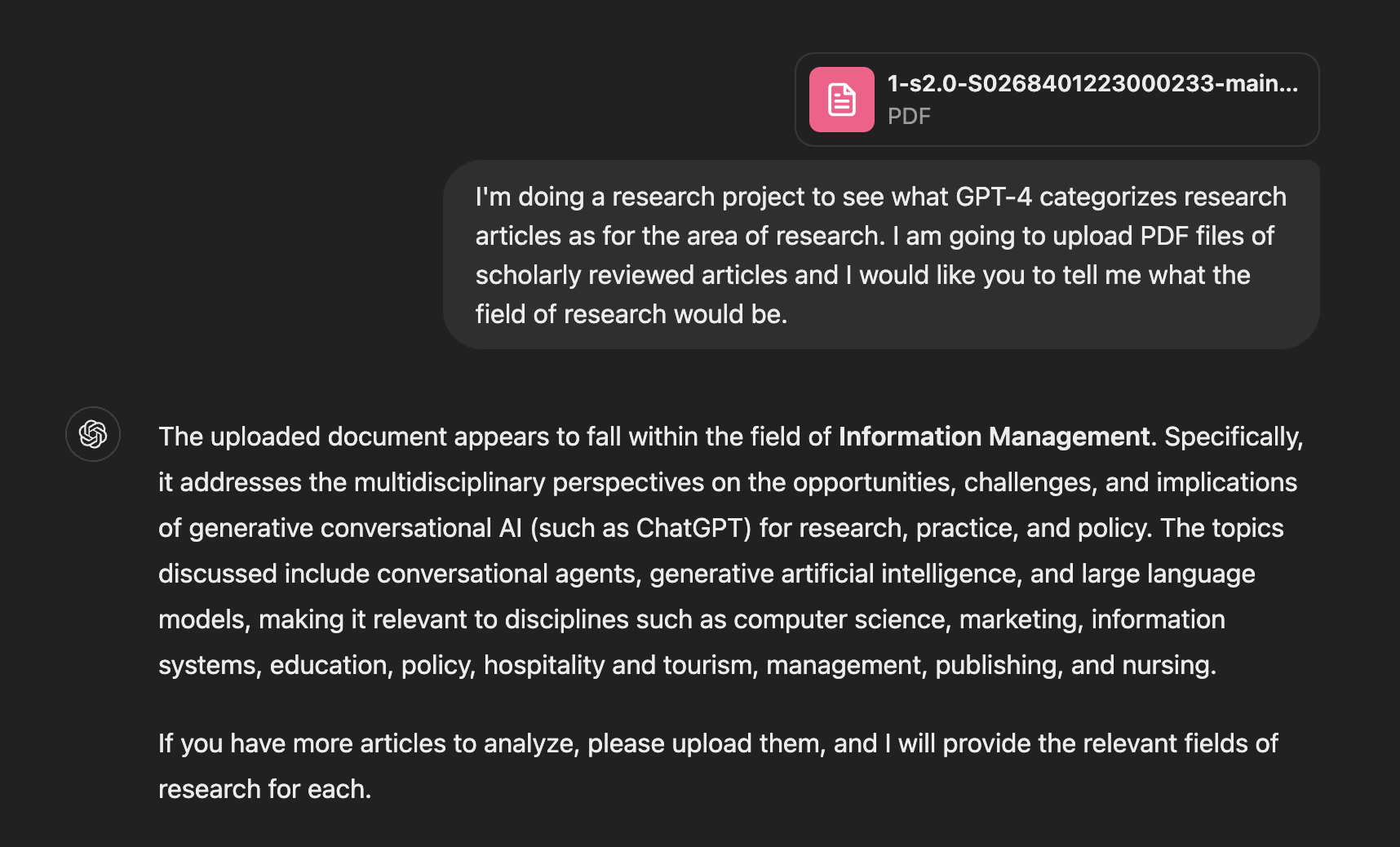

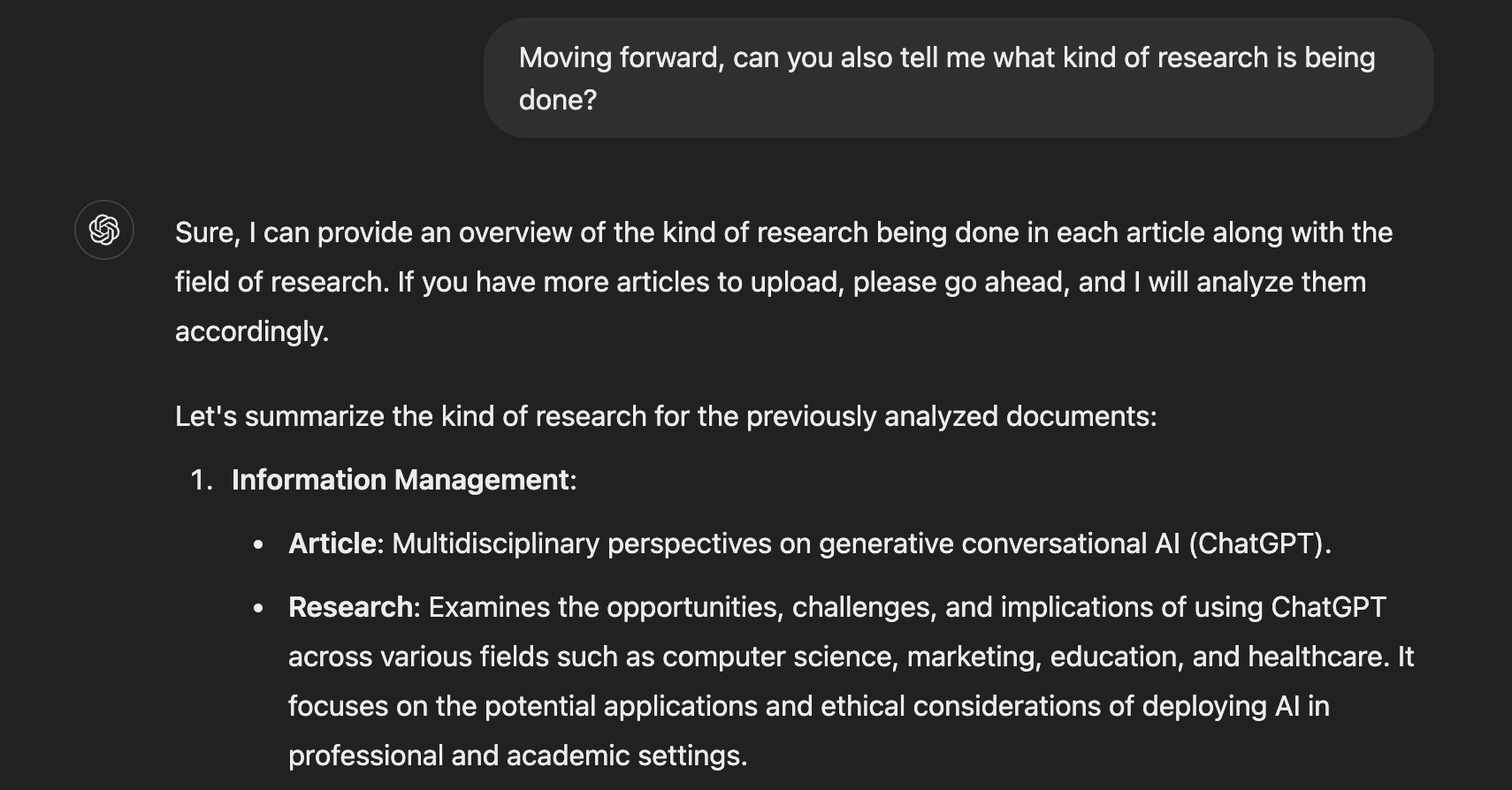
**

**Section 4. Bar graph of the frequency of each journal included in the top 100 cited list.**


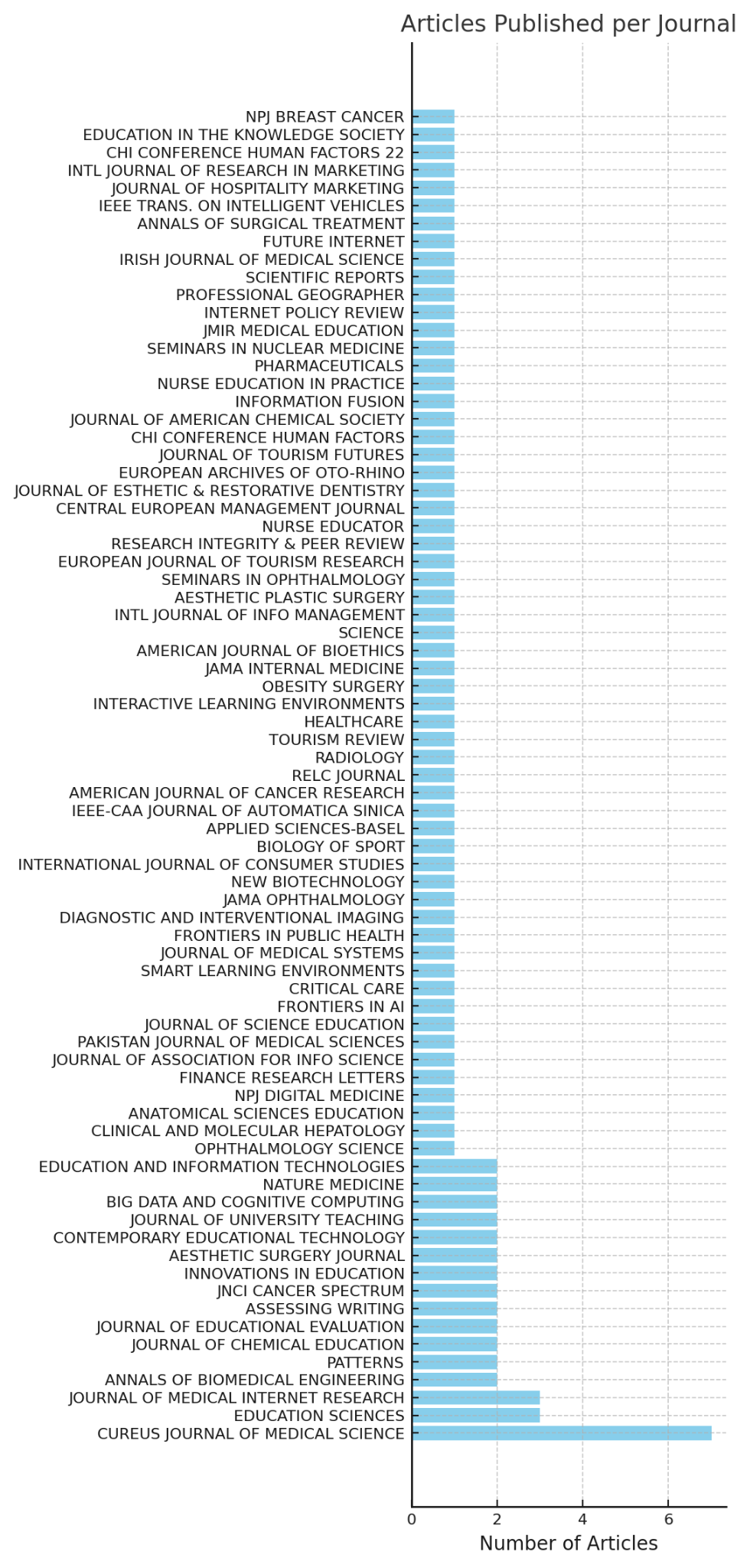


**Section 5. Summary of Medicine Articles**

| **Article Name** | **Article Type** | Times Cited (all databases) | Quality Assessment in Diagnosis / Medical Reasoning / Medical Knowledge | Quality Assessment in Research | Limitations of ChatGPT | Uses in Clinical Medicine | Uses in Research | Ethics | Medical Education |
| --- | --- | --- | --- | --- | --- | --- | --- | --- | --- |
| Sinha et al., 2023 | Quantitative Research | 54 | 1 |  |  |  |  |  |  |
| Alkaissi et al., 2023 | Qualitative Case Study | 227 | 1 |  | 1 | 1 | 1 |  |  |
| Das et al., 2023 | Quantitative Research | 29 | 1 |  | 1 |  |  |  |  |
| Salvagno et al., 2023 | Quantitative Research | 148 |  |  | 1 | 1 | 1 | 1 | 1 |
| Fatani, 2023 | Literature Review | 28 |  |  | 1 |  | 1 |  |  |
| Khan et al., 2023 | Conceptual Analysis | 125 |  | 1 | 1 | 1 |  | 1 | 1 |
| Ayers et al., 2023 | Mixed-Methods | 269 | 1 |  | 1 | 1 |  |  |  |
| Athaluri et al., 2023 | Quantitative Research | 35 |  | 1 | 1 | 1 | 1 | 1 |  |
| Waisberg et al., 2023 | Qualitative Research | 28 | 1 |  | 1 | 1 |  |  |  |
| Bhattacharyya et al., 2023 | Quantitative Research, Observational Study | 30 |  | 1 | 1 | 1 | 1 |  |  |
| Temsah et al., 2023 | Literature Review | 44 |  |  | 1 | 1 | 1 | 1 | 1 |
| Majovsky et al., 2023 | Case Study | 37 |  | 1 | 1 | 1 | 1 | 1 |  |
| Sallam, 2023 | Systematic Review | 355 |  |  | 1 | 1 | 1 | 1 | 1 |
| Gao et al., 2023 | Experimental Research | 84 |  | 1 | 1 | 1 | 1 |  |  |
| Cascella et al., 2023 | Qualitative Research | 173 | 1 | 1 | 1 | 1 | 1 | 1 |  |
| Choudhury et al., 2023 | Quantitative Research | 28 |  |  | 1 | 1 |  | 1 |  |
| Liu, S et al., 2023 | Mixed-Methods | 48 | 1 |  | 1 | 1 |  |  |  |
| Liu, J et al., 2023 | Review | 34 |  |  | 1 | 1 |  |  |  |
| Thirunavularasu et al., 2023 | Literature Review | 105 |  |  | 1 | 1 | 1 | 1 | 1 |
| Yeo et al., 2023 | Quantitative Research | 79 | 1 |  | 1 | 1 |  |  |  |
| Nature Medicine, 2023 | Editorial, Conceptual Analysis | 79 |  |  | 1 | 1 |  |  |  |
| Antaki et al., 2023 | Quantitative Research | 76 | 1 |  | 1 |  |  |  |  |
| Lecler et al., 2023 | Synthesis | 75 |  |  | 1 | 1 | 1 | 1 |  |
| Vaishya et al., 2023 | Literature Review | 65 | 1 | 1 | 1 | 1 | 1 |  |  |
| Mihalache et al., 2023 | Empirical Research |  | 1 |  |  |  |  |  | 1 |
| Skyler et al., 2023 |  |  | 1 |  | 1 | 1 |  |  |  |
| Hopkins et al., 2023 | Qualitative Research | 55 | 1 |  | 1 |  |  |  |  |
| Dergaa et al., 2023 | Literature Review | 55 |  | 1 | 1 |  | 1 | 1 | 1 |
| Huang and Tan, 2023 | Conceptual Analysis | 49 |  |  | 1 |  | 1 | 1 |  |
| Bhayana et al., 2023 | Qualitative Research | 48 | 1 |  | 1 |  |  |  | 1 |
| Samaan et al., 2023 | Quantitative Research | 44 | 1 |  | 1 | 1 |  |  |  |
| Singh et al., 2023 | Exploratory Research | 35 | 1 |  | 1 | 1 |  |  |  |
| Xie et al., 2023 | Quantitative Research | 34 | 1 |  | 1 | 1 |  |  |  |
| Gupta et al., 2023 | Quantitative Research | 33 |  | 1 |  |  | 1 |  |  |
| Eggman et al., 2023 | Literature Review | 32 |  |  | 1 | 1 |  | 1 | 1 |
| Hoch et al., 2023 | Quantitative Research | 31 | 1 |  | 1 |  |  |  | 1 |
| Humar et al., 2023 | Quantitative Research | 29 | 1 |  | 1 | 1 |  |  | 1 |
| Dave et al., 2023 | Conceptual Analysis | 133 |  |  | 1 | 1 | 1 | 1 | 1 |
| Oh et al., 2023 | Quantitative Research | 28 | 1 |  |  | 1 |  |  | 1 |
| Teixeira de Silva, 2023 | Conceptual Analysis | 28 |  |  | 1 |  | 1 |  |  |
| Cohen, 2023 | Opinion | 28 |  |  |  |  |  | 1 |  |
| Currie et al., 2023 | Literature Review | 26 |  |  | 1 | 1 | 1 | 1 | 1 |
| Sorin et al., 2023 | Retrospective Analysis | 26 | 1 |  | 1 | 1 |  | 1 |  |

**Section 6. Summary of Education Articles**

| **Article** | **Article Type** | **Times Cited (all data bases)** | **Privacy** | **Ethics** | **Negative effects on students** | **Solutions for AI Concerns** | **Quality assessment** | **Capabilities of AI** | **Uses for students** | **Uses for Educators** | **Limitations** |
| --- | --- | --- | --- | --- | --- | --- | --- | --- | --- | --- | --- |
| Fergus et al., 2023 | Exploratory Research | 44 |  | 1 |  |  | 1 | 1 |  |  | 1 |
| Emenike and Emenike, 2023 | Exploratory Research | 29 |  | 1 | 1 |  |  | 1 | 1 | 1 | 1 |
| Farrokhnia et al., 2023 | Literature Review | 97 |  | 1 | 1 | 1 | 1 | 1 | 1 | 1 |  |
| Perkins, 2023 | Conceptual Analysis | 78 |  | 1 |  | 1 |  |  |  |  |  |
| Huh, 2023 | Quantitative Research | 105 |  |  |  |  | 1 |  |  |  |  |
| Halaweh, 2023 | Conceptual Analysis | 48 |  | 1 | 1 | 1 |  | 1 | 1 |  |  |
| Cotton et al., 2024 | Conceptual Analysis | 177 |  | 1 |  | 1 |  |  | 1 | 1 | 1 |
| Su et al., 2023 | Conceptual Analysis | 26 |  | 1 |  | 1 |  |  | 1 | 1 | 1 |
| Cooper, 2023 | Exploratory Research; Performance Assessment | 99 |  | 1 |  |  | 1 | 1 |  |  | 1 |
| Thurzo et al., 2023 | Literature Review | 49 |  | 1 |  | 1 |  |  | 1 | 1 |  |
| Takagi et al., 2023 | Qualitative Research | 28 |  |  |  |  | 1 | 1 |  | 1 | 1 |
| Crawford et al., 2023 | Opinion; Commentary | 52 |  | 1 |  | 1 |  |  | 1 | 1 | 1 |
| Adiguzel et al., 2023 | Review | 47 | 1 | 1 |  | 1 |  | 1 | 1 | 1 | 1 |
| Grassni et al., 2023 | Literature Review; Conceptual Analysis | 26 |  | 1 | 1 | 1 |  |  |  | 1 | 1 |
| Garcia-Peñalvo, 2023 | Literature Review | 38 |  | 1 |  | 1 |  |  |  |  | 1 |
| Barrot, 2023 | Conceptual Analysis | 27 |  | 1 |  | 1 |  |  | 1 | 1 | 1 |
| Lo, 2023 | Literature Review | 129 |  |  |  |  | 1 | 1 | 1 | 1 | 1 |
| Tlili et al., 2023 | Qualitative Case Study | 165 | 1 | 1 |  | 1 | 1 |  | 1 | 1 | 1 |
| Jeon and Lee, 2023 | Qualitative Research; Exploratory Research | 33 |  |  |  |  |  | 1 | 1 | 1 |  |
| Lee, 2023 | Conceptual Analysis | 81 | 1 | 1 |  | 1 |  |  | 1 | 1 | 1 |
| Strzelecki, 2023 | Quantitative Research | 44 |  |  |  |  |  |  | 1 |  |  |
| Yan, 2023 | Exploratory Research | 48 |  | 1 | 1 |  | 1 |  | 1 |  |  |
| Rahman and Watanobe, 2o23 | Mixed- Methods | 51 |  | 1 | 1 | 1 | 1 | 1 | 1 | 1 | 1 |
| Hosseini and Horbach, 2023 | Conceptual Analysis | 33 |  | 1 |  | 1 |  | 1 |  |  | 1 |
| Kohnke et al., 2023 | Literature Review | 48 |  | 1 |  | 1 |  | 1 | 1 | 1 | 1 |
| Sun and Hoelscher, 2023 | Conceptual Analysis | 33 |  | 1 | 1 | 1 |  | 1 | 1 | 1 | 1 |

**Section 7. Summary of Technology and Information Sciences Articles**

| **Article** | **Article Type** | **Times Cited (all data bases)** | **Technical uses of ChatGPT** | **Limitations** | **Quality Assessment** | **Privacy Concerns (User Data)** | **Ethics, Privacy Concerns** | **Future** |
| --- | --- | --- | --- | --- | --- | --- | --- | --- |
| Du et al., 2023 | Exploratory Research; Conceptual Analysis | 30 | 1 | 1 |  | 1 |  |  |
| Roumeliotis and Tselikas, 2023 | Literature Review | 31 | 1 | 1 |  |  |  |  |
| Kocon et al., 2023 | Case Study; Mixed Methods | 30 | 1 | 1 | 1 |  | 1 |  |
| Vaithilingam et al., 2022 | Mixed Methods | 36 | 1 | 1 |  |  |  | 1 |
| Anders, 2023 | Opinion | 28 |  |  |  |  |  | 1 |
| Hassani and Silva, 2023 | Literature Review | 48 | 1 | 1 |  | 1 | 1 |  |
| Chatterjee and Dethlefs, 2023 | Opinion | 37 | 1 |  |  |  |  | 1 |
| Taecharungroj, 2023 | Qualitative Research | 70 |  |  |  |  |  | 1 |
| Wu et al., 2022 | Qualitative Case Study | 36 | 1 |  |  |  |  | 1 |
| Wu et al., 2023 | Synthesis | 47 | 1 |  |  |  | 1 | 1 |
| Noy and Zhang, 2023 | Experimental Research | 38 | 1 |  | 1 |  |  | 1 |
| Biswas, 2023 | Exploratory Research | 36 | 1 | 1 |  |  |  |  |
| Lund et al., 2023 | Overview | 92 |  | 1 |  |  | 1 |  |
| Dwivedi et al., 2023 | Opinion | 386 | 1 | 1 |  | 1 | 1 | 1 |

**Section 8. Thematic categorization of journals and authors by ChatGPT.**

**
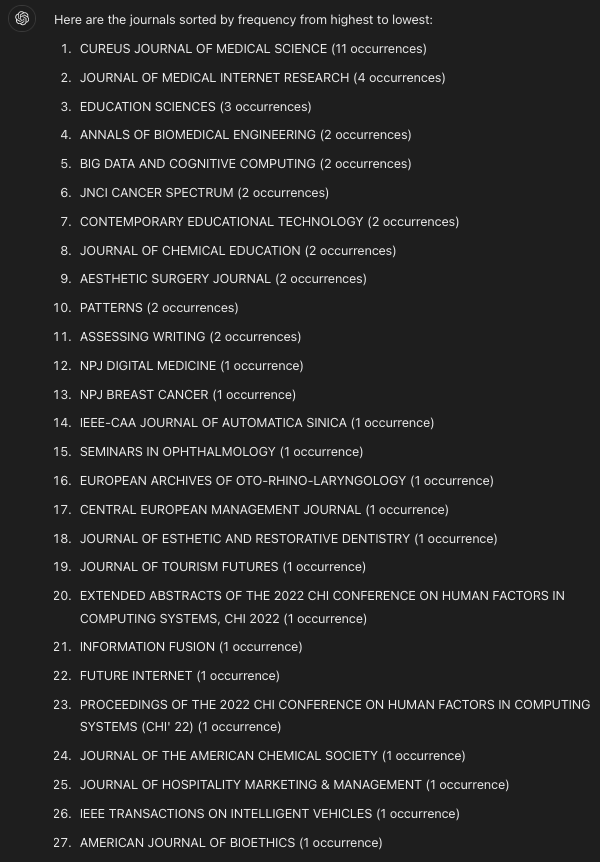

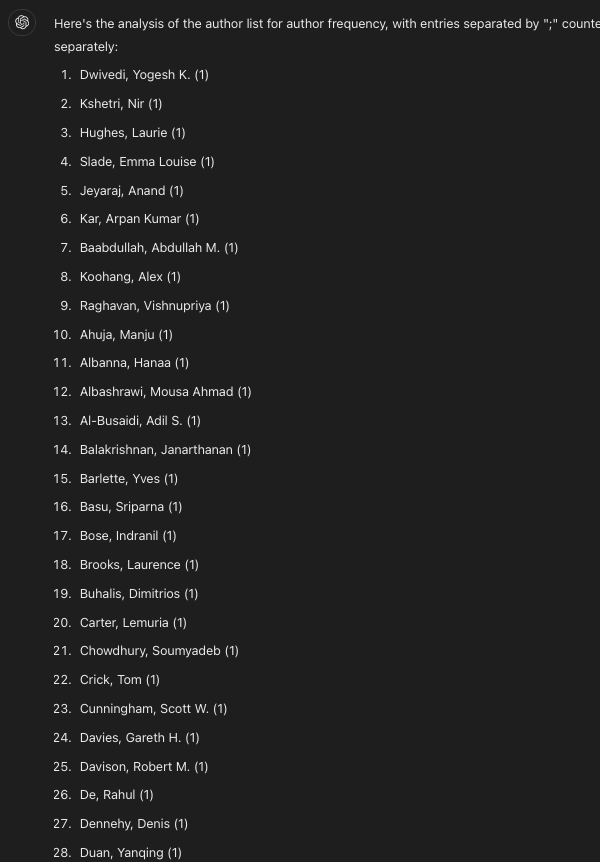
**
